# Spontaneous Hematoma of the Rectus Sheath: Urgent Embolization with Squidperi Liquid Embolic Device

**DOI:** 10.1155/2017/3420313

**Published:** 2017-07-13

**Authors:** Pierluca Torcia, Umberto G. Rossi, Silvia Squarza, Maurizio Cariati

**Affiliations:** ASST Santi Paolo and Carlo, San Carlo Borromeo Hospital, Department of Diagnostic Science, Radiology and Interventional Radiology Unit, Via Pio II 3, 20153 Milano, Italy

## Abstract

We treated a 78-year-old female affected by nontraumatic spontaneous rectus sheath hematoma. We decided to perform the embolization with the new liquid agent Squidperi. Complete exclusion of the bleeding vessel was obtained without complications. Its use should be considered for treatment of nontraumatic rectus sheath hematoma.

## 1. Introduction

Rectus sheath hematoma is a blood collection in the sheath of the anterior rectus muscle caused by epigastric artery damage or tearing of one or more muscle fibers [1].

Nontraumatic spontaneous hematoma, generally due to anticoagulation therapy, is not considered a critical condition, especially in elderly patients. Nevertheless, despite correct medical treatment, some patients with continuous and/or consistent bleeding, presenting a large hematoma with a consistent hypovolemia, request a more radical handling. Percutaneous management by selective catheters and embolization of the bleeding vessel is considered the gold standard for nonmedical therapy [2].

We describe a case of nontraumatic spontaneous hematoma treated with Squidperi, a new formulation available for embolization procedures.

## 2. Case Report

**Figure 1:**
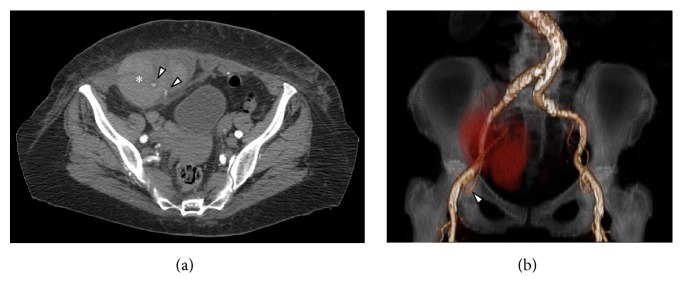
(a) MD-CT axial view demonstrates the presence of a haematoma into the right rectus sheath (*∗*) with two spots of active bleeding from distal collaterals of the right inferior epigastric artery (arrowheads). (b) MD-CT coronal Volume Rendering Technique image with right inferior epigastric artery reconstruction (arrowhead) and the haematoma into the right rectus sheath as a red shadow.

A 78-year-old female, with acetylsalicylic acid therapy for a previous stroke, presented to our emergency department for a huge and tender right lower abdominal mass. She denied any traumatic event. Her haemoglobin level was 7.1 g/dL. She underwent urgent MultiDetector Computed Tomography (MD-CT) that demonstrated a 12 × 9 cm right rectus sheath haematoma extending into the pelvis with two active contrast extravasation (Figures 1(a) and 1(b)).

**Figure 2:**
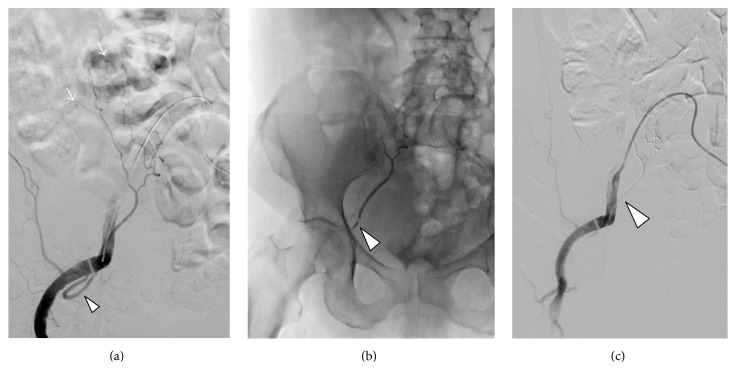
(a) Selective right iliac angiography confirmed the two spots of active bleeding (arrows) from distal collaterals of the right epigastric artery (arrowhead). (b) Fluoroscopic control with the right epigastric artery (arrowhead) filled by Squidperi 18 liquid embolic material. (c) Final selective right iliac angiography that confirms the embolization of the right epigastric artery (arrowhead) filled by Squidperi 18 liquid embolic material as a grey shadow.

After multidisciplinary agreement, the patient underwent urgent Digital Subtraction Angiography (Figure 2(a)) that confirmed the two spots of active bleeding from distal collaterals of the right epigastric artery. After coaxial superselective catheterization of the right epigastric artery with Terumo Progreat microcatheter 2.7 Fr (Terumo, Tokyo, Japan) and a previous shaking for at least 15 minutes of the liquid embolic device Squidperi 18 (Emboflu, Switzerland), the two types of active bleeding and the right epigastric artery were embolized (Figure 2(b)). The final DSA control confirms the complete embolization of the right epigastric artery (arrowhead) with absence of active bleeding (Figure 2(c)).

**Figure 3:**
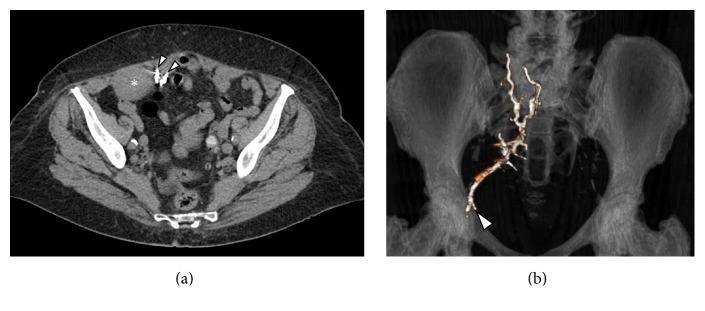
(a) MD-CT axial view at one month demonstrates the reduction of the haematoma into the right rectus sheath (*∗*) with two hyperdense artefact due to the Squidperi liquid embolic material. (b) MD-CT coronal Volume Rendering Technique image at three months with right epigastric artery reconstruction (arrowhead) filed by Squidperi 18 liquid embolic material.

The postoperative course was uneventful. Patient was discharged on day 3 with an increased haemoglobin level (10.4 g/dL). Early follow-up at 3 months after embolization (including clinical observation, assessment of abdominal symptoms, and MD-CT) demonstrated no clinical complications, reduction of the haematoma into the right rectus sheath, and persistence as hyperdense image referred to Squidperi liquid embolic material (Figures 3(a) and 3(b)).

## 3. Discussion

Nontraumatic spontaneous rectus sheath hematoma is a rare condition generally treated with medical therapy. When necessary, while surgery evacuation and bonding of concerned bleeding vessels were in the past the most used procedures, the endovascular percutaneous treatment is the gold standard at the present. Selective catheterization, the use of microcatheters, and the versatility of embolization material allow a correct management in any type of situation [3].

MD-CT is generally used to reveal the real size and extension and to depict the existence of the active bleeding in the contest of hematoma. Selective arteriography instead is the most useful image technique to identify the presence and location of the bleeding; it provides information on the involved artery branches that support the bleeding and its exact location [2].

The use of a microcatheter allows stopping the bleeding in a precise and selective way reducing eventual dispersion of permanent embolization material or mechanical material. Embolization is generally performed by coils, which allow a more precise handling at the given location, immediately stopping the bleeding, especially when little vessels are concerned [4].

Other disposable materials, even if not frequently used for embolization, are cyanoacrylate glue with percutaneous injection or Amplatzer Vascular Plug [5, 6].

Before the commercialization of Squid, the only available liquid formulation for embolization procedures was Onyx, produced by EV3 (Neurovascular, Irvine, CA, USA).

Squidperi is a nonadhesive liquid embolic agent composed of EVOH (ethylene vinyl alcohol) copolymer dissolved in DMSO (dimethyl sulfoxide) and suspended micronized tantalum powder. Generally it is indicated for embolization of lesions in the peripheral vasculature, including arteriovenous malformations and hypervascular tumors. Two forms are commercially available, Squid 12 and Squid 18.

First experience with Squid was described in 2014 by Akmangit et al. They used Squid for treatment of arteriovenous malformations, arteriovenous fistula, aneurysms, and tumors [7].

In 2014 Salik et al. reported the use of Squid 12 to perform concomitantly transarterial and transvenous embolization of a pelvic arteriovenous malformation [8, 9].

Recently Squid 18 has been used for the management of a skull base osseous fistula [10].

To our knowledge, there are no reports in literature regarding embolization of a rectus sheath hematoma with Squid, especially in geriatric patients. In our experience, principal limit of its use may be the time requested to prepare the formulation (about 20 minutes), especially if emergency treatment is required. On the other hand, Squid turned out to be a safe and effective means of obtaining the complete embolization of the bleeding vessel. We suggest its use as liquid embolic agent for the treatment of nontraumatic spontaneous rectus sheath hematoma.
